# Perceptions of mental health stigma toward diagnostic labels among health professionals and mental health users: a Delphi study

**DOI:** 10.3389/fpsyg.2026.1863313

**Published:** 2026-08-07

**Authors:** María del Pilar Ferrer-Martínez, María M. Hurtado, Elena Navarro-González, Sofia Amaoui, Yolanda Mejías-Martín, Carlos Aguilera-Serrano, Celia Martí-García

**Affiliations:** 1 Departamento de Enfermería, Facultad de Ciencias de la Salud, Universidad de Málaga, Málaga, Spain; 2 Hospital Regional Universitario de Málaga, UGC de Salud Mental, Málaga, Spain; 3 Department of Personality, Assessment, and Psychological Treatment, University of Granada, Granada, Spain; 4 Department of Psychology, University of Innsbruck, Innsbruck, Austria; 5 Hospital Universitario Virgen de las Nieves, Granada, Spain

**Keywords:** Delphi technique, diagnosis, mental disorders, mental health, social stigma

## Abstract

**Background:**

The stigma associated with mental disorders influences help-seeking behavior, quality of care, and recovery outcomes. Although diagnostic labels are essential for clinical communication and research, they may also evoke negative social perceptions that contribute to discrimination and self-stigma. Accordingly, the present study aimed to evaluate the perceived degree of stigmatization of a set of diagnostic labels for mental disorders, and secondarily to explore their perceived sex-based applicability.

**Methods:**

A total of 61 terms, including diagnostic labels derived from the DSM-5 were linguistically transformed into adjectival forms and evaluated using a Delphi methodology. The study was conducted between March and July 2025 and consisted of three sequential rounds. A multidisciplinary panel comprising 36 health professionals and 14 mental health service users rated each label on a 9-point Likert-type scale according to its perceived stigmatizing potential. Consensus was assessed following the criteria established by the RAND Corporation, using percentile distributions and interquartile ranges. Ratings on whether each label was perceived as more commonly associated with men, women, or both equally were also collected.

**Results:**

A total of three Delphi rounds were conducted with the aim of achieving the highest possible level of consensus among participants. Of the 61 labels evaluated, 20 reached consensus (32.78%). Among these, 95% were classified as highly stigmatizing and 5% as low in stigma, while none reached consensus for a moderate level of stigmatization. In terms of sex-based applicability, 51% of the labels were perceived as applicable to both sexes, 31% as more applicable to men, and 18% as more applicable to women.

**Conclusion:**

Results showed that only a limited number of labels reached consensus regarding their stigmatizing level, highlighting the subjective and complex nature of stigma surrounding diagnostic language.

## 1 Introduction

Mental disorders (MDs) represent a major global public health challenge, with substantial clinical and social impact (Dattani et al., 2023; Jin et al., 2021). Beyond their economic cost, and the associated symptom burden, individuals with MDs frequently experience stigma and discrimination, which negatively affect help-seeking, treatment engagement, and recovery (Awan et al., 2025; Shalaby et al., 2025).

Stigma has been conceptualized as a social process involving labeling, stereotyping, separation, and status loss (Goffman, 1970; Link and Phelan, 2001). In mental health contexts, stigmatizing attitudes—including those observed in healthcare settings—can compromise communication, shape recovery expectations, and worsen outcomes (Haque et al., 2021; Parker, 2019; Valery and Prouteau, 2020). This inadequate care contributes to increased morbidity and mortality among affected individuals, not only through delays in the detection and treatment of comorbid physical conditions, but also through the direct detrimental effects of stigma on mental health (Daré et al., 2019). Diagnostic labels play a central role in this process. While indispensable for clinical communication and treatment planning (Harvey et al., 2012), they may also activate negative social representations and contribute to both public stigma and self-stigma (Ben-Zeev et al., 2010; Corrigan, 2007).

While some negative stereotypes may stem from symptoms intrinsic to the disorder, most arise from social stigma (Corrigan and Wassel, 2008). Thus, MDs are not only individual clinical conditions, but also socially constructed categories shaped by cultural, economic, and interpersonal factors that influence how they are perceived and stereotyped (Corrigan et al., 2018). Stigma is therefore not an isolated phenomenon, but a social construction reinforced by cultural and structural norms (Link and Phelan, 2001).

Language plays a central role in shaping these perceptions. Common expressions such as “crazy” or “losing one's mind” appear frequently in the media and everyday discourse, often portraying mental illness as dangerous or abnormal (Wilson et al., 2000). Words associated with MDs may therefore acquire social meanings that extend beyond their clinical definitions, contributing to discrimination, social devaluation, and avoidance of help-seeking (Corrigan, 2004; Link et al., 2004). Understanding how these labels are socially perceived is thus relevant for both stigma research and mental health practice (Link and Phelan, 2001).

Beyond their classificatory function, diagnostic labels are not neutral descriptors but linguistically and socially embedded terms that can acquire affective meaning through learning and social experience. From the perspective of Lang's bioinformational model, words can function as affective stimuli capable of activating emotional representations (Bradley and Lang, 1999; Lang et al., 1990). Because stigma forms part of the social meaning attached to many diagnostic labels, characterizing their perceived stigmatizing potential represents an important preliminary step for future research, including studies examining emotional responses to diagnostic language.

Importantly, the prevalence, expression, and social interpretation of MDs are not uniform across the population, showing consistent differences according to sex. Women are more frequently diagnosed with anxiety, depression, and psychological distress, whereas men more frequently experience disorders related to substance use or impulse control (Maestre-Miquel et al., 2021; McLaughlin et al., 2014). These differences should be interpreted considering potential gender-related variations in symptom expression, diagnostic practices, and help-seeking. Concerning the latter, stigma plays a major role in this reluctance, as fear of negative judgment often delays both initial help-seeking and follow-up care (Amatya et al., 2018; Awan et al., 2025). Gender stereotypes may further exacerbate this situation, with men tending to hold more stigmatizing attitudes toward mental illness and therefore seeking less professional help (Atienza-Carbonell et al., 2024; Kaitz et al., 2022; McKenzie et al., 2022).

Additionally, recent literature shows that these gender stereotypes influence how MDs are conceptualized, reinforcing existing stigma. Some diagnostic labels are predominantly associated with men, such as those related to paraphilias, addictions, or antisocial behaviors, while others—like eating disorders or certain sexual dysfunctions—are more frequently attributed to women. In this sense, it has been observed that disorders linked to masculinity tend to generate higher levels of stigmatization (Boysen, 2017; Mostoller and Mickelson, 2024). Thus, these sex-based differences extend beyond epidemiological patterns, encompassing social meanings, stereotypes, and culturally shared representations associated with MDs.

As discussed above, stigma associated with MDs constitutes a major social and public health concern, contributing to health inequalities and negatively affecting help-seeking, treatment engagement, and recovery (Lien et al., 2019; Thornicroft et al., 2022).

Despite extensive research on mental health stigma, relatively little evidence is available regarding the perceived stigmatizing potential of many specific diagnostic labels, particularly within the Spanish linguistic context. Consequently, the present study was conceived as an exploratory investigation aimed at characterizing these labels and identifying areas of consensus and disagreement regarding their perceived stigmatization.

Accordingly, the primary aim of this study was to evaluate, using the Delphi approach, the perceived degree of stigmatization of mental health–related labels, including both DSM-5–derived diagnostic terms and commonly used stigma-related expressions, as judged by health professionals and mental health service users. As a secondary objective, we explored the perceived sex-based applicability of these labels.

This study constitutes a foundational phase of the Stigmotion project, providing the initial characterization of diagnostic labels required for their subsequent use as affective linguistic stimuli in experimental research. Pre-registration and study materials are publicly available on the Open Science Framework platform (https://osf.io/sg7fb?view_only=893cde3d49a84e499747463572e64425).

## 2 Method

### 2.1 Design

The present study followed a quantitative Delphi design focused on the expert- and user-based evaluation of diagnostic labels in terms of perceived stigmatization and sex-based applicability, prior to their affective characterization within emotional models such as that proposed by Lang. Diagnostic labels were transformed into adjectival forms and evaluated using a structured Delphi process (Fitch, 2001).

The study was conducted between March and July 2025 and consisted of three sequential rounds.

### 2.2 Participants and sampling

A total of 76 potential participants were invited to take part in the study, including professionals from diverse health-related and social disciplines (*n* = 53) and mental health service users (*n* = 23). A purposive sampling strategy was used to incorporate complementary perspectives derived from professional practice and lived experience.

The inclusion criteria for professionals (hereafter referred to as health professionals) required: (a) being a professional with training and/or regular contact with individuals with a mental health diagnosis, including (but not limited to) primary care physicians, nurses, mental health nurses, clinical psychologists, psychologists, psychiatrists, social workers, and occupational therapists; (b) holding a professional and/or academic background related to mental health and; (c) having at least 3 years of professional experience in roles involving mental health care or support.

In the case of mental health service users, inclusion criteria required participants: (a) to be at least 18 years old; (b) to have a formal mental health diagnosis and at least 1 year of lived experience with the condition and (c) to have sufficient cognitive and functional capacity to participate autonomously in all phases of the Delphi process. For recruitment, the research team employed a purposive identification process in collaboration with mental health professionals familiar with potential participants.

### 2.3 Term selection and adjectival transformation

The process began with the selection of diagnostic labels based on the DSM-5 classification. The research team— composed of professionals with formal training and extensive clinical and research experience in the field of mental health—reviewed 70 diagnoses and selected 44 labels, prioritizing those with the greatest clinical relevance and frequency in professional practice.

Importantly, two conceptually distinct types of terms were included in the stimulus set: (a) diagnostic labels derived from DSM-5 categories (e.g., “schizophrenic”, “bipolar”), and (b) colloquial or stigma-related expressions (e.g., “crazy”, “unbalanced”), which do not correspond to formal diagnostic categories but are widely used in everyday language and stigma-related discourse (Angermeyer and Dietrich, 2006; Corrigan, 2000; Link and Phelan, 2001; Pescosolido et al., 2010; Thornicroft et al., 2007). The research team decided to include both type of terms since, although these categories are conceptually distinct, they function as linguistic representations associated with mental health disorders and may differentially contribute to stigma. Nonetheless, a distinction must be made between diagnostic categories, understood as formal clinical constructs defined by classification systems such as the DSM-5, and their linguistic representations (e.g., adjectival labels such as “schizophrenic” or “bipolar”). The present study focuses on the latter, namely the language-based expressions of diagnostic concepts, which may carry social meanings that extend beyond their clinical definition.

All terms were linguistically transformed into adjectival forms to create evaluable stimuli. In the first round, each term was presented in both masculine and feminine forms. As a result, the stimulus set for the first round comprised a total of 46 terms, including both DSM-5–derived diagnostic labels and colloquial expressions. For consistency, the complete set of stimuli will hereafter be collectively referred to as diagnostic labels, acknowledging their heterogeneity.

### 2.4 Instruments

The final survey consisted of two sections. The first section collected sociodemographic and, when applicable, professional data, including information related to participants' experience with the diagnosis (e.g., age, sex, education, professional background, time since diagnosis, and availability to complete all Delphi rounds). The second section focused on assessing the degree of stigmatization of the diagnostic labels and their perceived applicability according to sex.

In each round, participants rated the adjectival labels using a 9-point Likert-type scale (1 = “none,” 9 = “maximum”) to determine their level of stigmatization. Perceived sex-based applicability was assessed separately using a nominal response option in which participants selected whether each label was more commonly associated with men, women, or both. Participants could also provide comments or suggestions regarding each term and potential gender-related considerations. Because this study was conducted in Spain, where adjectives have grammatical gender (masculine/feminine), the research team agreed that in cases without a clear majority, the option with the highest percentage would be selected. When the majority indicated “both,” the sex with the greater relative weight in percentages was chosen.

Prior to data collection, the questionnaire was tested by the research team to ensure clarity, comprehensibility, and linguistic accuracy of the items.

### 2.5 Procedure

Participants who met the inclusion criteria were contacted by email and invited to complete an online survey. Each round remained open for 1 week, during which reminder emails were sent to participants.

At the end of each round, participants received a report summarizing the results. In the first and second rounds, this report also included an invitation to continue participating in the next phase.

#### 2.5.1 Rounds

**Figure 1:**
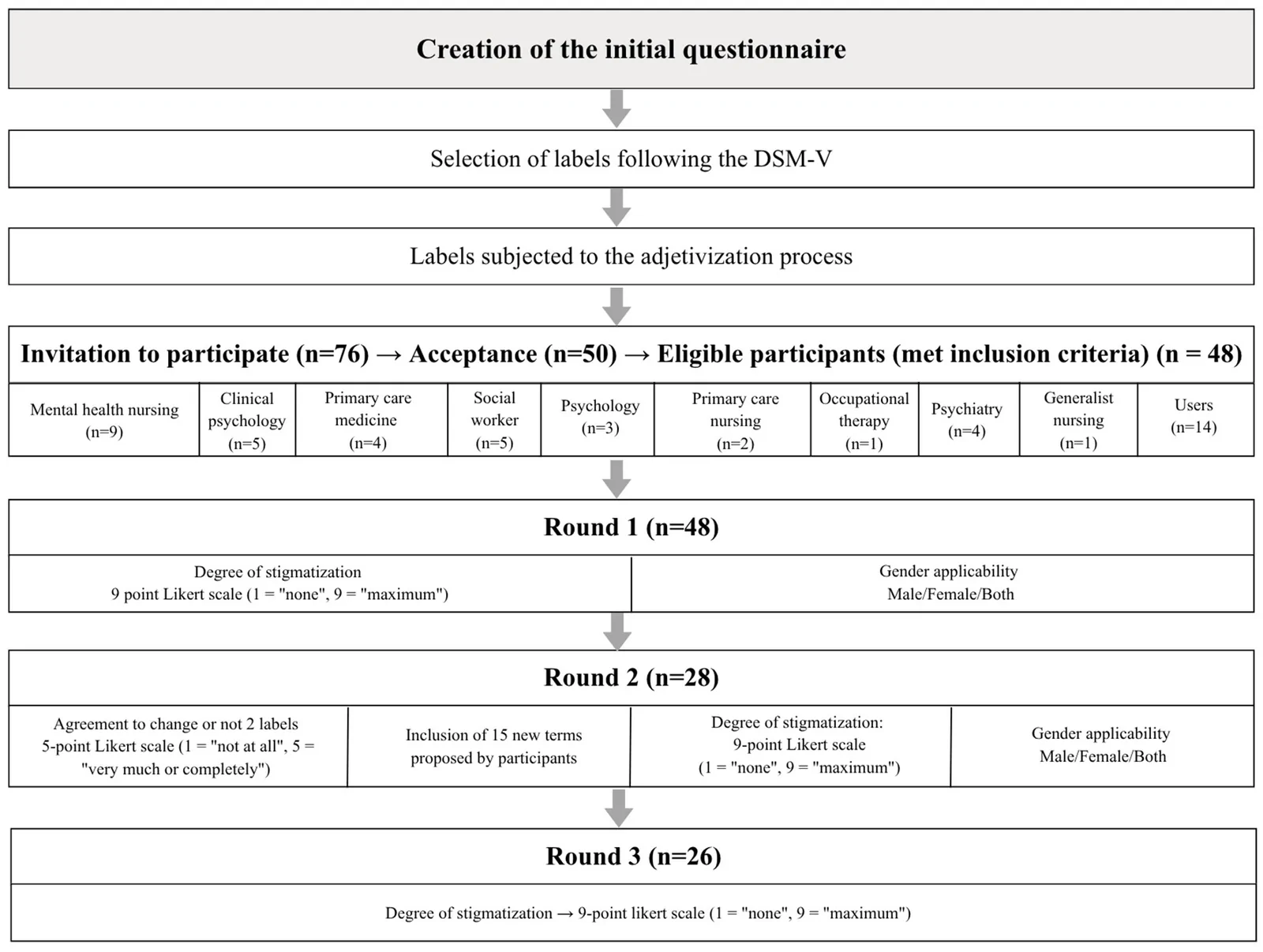
Flow diagram of the Delphi process. Participant flow across the three Delphi rounds and summary of the evaluations performed at each stage of the consensus-building procedure.

The study concluded after the third round. In each round, participants rated the diagnostic labels and provided qualitative comments where necessary. In subsequent rounds, terms that had not reached consensus were re-evaluated. Based on participants' feedback, the research team also revised the list by adding new terms and removing those deemed inappropriate (see Figure 1).

Consistent with the exploratory aims of the study, the set of diagnostic labels was iteratively refined across rounds based on participants' qualitative feedback and research team consensus. Accordingly, this phase should be understood as a process of systematic characterization and refinement of diagnostic labels rather than the validation of a fixed stimulus set. Consensus was evaluated independently for each label and based exclusively on the rounds in which that label was presented.

### 2.6 Ethical considerations

The study was approved by the Biomedical Research Ethics Committee of Andalusia (SICEIA-2024-002595). All participants provided written informed consent prior to participation. Data were pseudo anonymized using coded identifiers accessible only to the research team. All procedures complied with the Declaration of Helsinki and the current legislation on personal data protection.

### 2.7 Data analysis

At the end of each round, the data were analyzed following the criteria established by the RAND Corporation (Fitch, 2001), which specify the use of the interquartile range (IQR) and percentile distribution as measures of agreement, disagreement, and consensus level. Given that the rating scale ranged from 1 to 9, consensus on a label being highly stigmatizing was defined as at least 75% of participants assigning it a score between 7 and 9. Conversely, if ratings were between 1 and 3, the label was considered to have low stigmatizing character, and scores between 4 and 6 indicated a moderate level of stigmatization. If the interquartile range exceeded a value of 3, disagreement among participants was assumed, and a new evaluation round was conducted for those items.

For the assessment of perceived sex-based applicability, responses were treated as nominal categorical data, with three possible options (men, women, or both). Consequently, agreement was evaluated using frequency distributions and percentages.

Group comparisons were performed using parametric or non-parametric tests, and categorical variables were analyzed using chi-square or Fisher's exact tests as appropriate.

Comparisons between health professionals and mental health service users were considered secondary and exploratory analyses. Consequently, their results should be interpreted as descriptive indicators of potential group differences rather than as confirmatory tests of predefined hypotheses. Effect size estimates were calculated to facilitate interpretation of the magnitude of observed differences.

## 3 Results

A total of 76 participants were invited to take part in the study, including 53 health professionals and 23 mental health service users. Of these, 50 participants completed the questionnaire in the first round. Two responses were subsequently excluded because the participants did not meet the predefined inclusion and exclusion criteria, resulting in a final sample of 48 participants for the analysis (34 health professionals and 14 service users).

The mean age of the sample was 46.06 years (SD = 10.80), with 64.58% identifying as women and 35.42% as men. No statistically significant differences were observed between health professionals and service users in sociodemographic variables, except for educational level (*p* = 0.002), which was significantly higher among health professionals (all other *p* > 0.05).

The professional qualifications of the health professionals were as follows: Mental health nursing (*n* = 9), Clinical psychology (*n* = 5), Primary care medicine (*n* = 4), Social work (*n* = 5), Psychology (*n* = 3), Primary care nursing (*n* = 2), Occupational therapy (*n* = 1), Psychiatry (*n* = 4), and General nursing (*n* = 1; See Table 1). Regarding professional activity, 7 participants worked primarily in research or teaching, 24 in clinical practice, and 3 combined both fields.

**Table 1:** Sociodemographic variables (*N* = 48).

Variable	Health professionals	Mental health service users	Total value/*n* (%) or mean (SD)	*p*
Participant profile	34 (70.83)	14 (29.17)		
Age	47.12 (10.05)	43.50 (12.45)	45.32 (11.19)	0.296
Sex				0.194
Female	20 (58.8)	11 (78.6)	31 (64.58)	
Male	14 (41.2)	3 (21.4)	17 (35.42)	
Education level				0.002
Primary education	0 (0)	1 (7.1)	1 (2.1)	
Secondary education	0 (0)	4 (28.6)	4 (8.3)	
Higher education (undergraduate)	15 (44.1)	2 (14.3)	17 (35.4)	
Doctorate or postgraduate studies	19 (55.9)	7 (50)	26 (54.2)	
Place of residence				0.411
Southern Spain	23 (67.6)	13 (92.8)	36 (75)	
Eastern Spain	6 (17.6)	1 (7.1)	7 (14.58)	
Central Spain	2 (5.9)	0 (0)	2 (4.16)	
Islands	3 (8.8)	0 (0)	3 (6.25)	
Primary area of residence				0.986
Rural area (fewer than 50,000 inhabitants)	2 (5.9)	1 (7.1)	3 (6.3)	
Peri-urban area (outskirts of the city)	5 (14.7)	2 (14.3)	7 (14.6)	
Urban area (city with more than 50,000 inhabitants)	27 (79.4)	11 (78.6)	39 (79.2)	
Time elapsed since receiving the diagnosis^a^				
Between 1 and 3 years		3 (21.43)	
Between 3 and 5 years				
More than 5 years		10 (71.43)		
Prefer not to answer		1 (7.14)		
Profession^b^				
Mental health nurse specialist	9 (26.5)			
General nurse	1 (2.9)			
Primary care nurse	2 (5.9)			
Psychologist	3 (8.8)			
Clinical psychologist	5 (14.7)			
Psychiatrist	4 (11.8)			
Primary care physician	4 (11.8)			
Occupational therapist	1 (2.9)			
Social worker	5 (14.7)			
Years of professional experience^b^				
Between 3 and 5 years	2 (5.9)			
Between 6 and 10 years	5 (14.7)			
More than 10 years	27 (79.4)			
Main professional role^b^				
Primarily academic/research	7 (20.6)			
Primarily clinical practice	24 (70.6)			
Both clinical practice and academic/research	3 (8.8)			
Type of employing institution^b^				
Public	32 (94.1)			
Private	1 (2.9)			
Patient association	1 (2.9)			
Frequency of working with patients (only clinical practice)				
Daily	26 (96.3)			
Occasionally	1 (3.7)			
Previous participation in any other Delphi study				
Yes			19 (39.6)	
No			29 (60.4)	

^a^Calculated based on 14 participants (service users only); ^b^Applicable only to those who participated as professionals.

Among the mental health service users, 11 were women and 3 were men. Regarding educational level, 50% held a doctorate or postgraduate degree, 14.29% had a university degree, 28.57% had completed secondary education, and 7.14% had primary education. The time elapsed since the diagnosis of a MD varied among participants: the majority (71.43%) had been diagnosed more than five years ago, 21.43% had been diagnosed between 1 and 3 years ago, and one participant indicated “prefer not to answer” (7.14%).

A total of three Delphi rounds were conducted with the aim of achieving the highest possible level of consensus among participants.

### 3.1 Round 1

In the first Delphi round, participants' evaluations revealed varying levels of stigmatization across the 46 diagnostic terms included. Seven labels reached consensus as being highly stigmatizing (e.g., *schizophrenic, crazy, heroin addict*, and *pyromaniac*), while one was rated as low in stigmatization (*apneic*). The remaining 38 labels did not achieve consensus.

Most labels were perceived as applicable to both sexes (56.52%; e.g., *crazy, amnesic, and depersonalized*). In contrast, 17.39% were predominantly associated with women (e.g., *anorexic* and *frigid*), and 26.09% with men (e.g., *pyromaniac*; see Table 2).

**Table 2:** Round 1 results (*N* = 48).

Label			Level of stigmatization				
	*M*	IQR	High (7–9)	Medium (4–6)	Low (1–3)	Consensus	Sex-related applicability
Agoraphobic (Agoraphobia)	4.75	3	15%	54%	31%	No	F^*^
Alcoholic (Alcohol Use Disorder)	7.10	3	67%	29%	4%	No	M
Amnesic (Dissociative Amnesia)	3.77	3	13%	35%	52%	No	F^*^
Anorexic (Anorexia Nervosa)	6.21	3	52%	38%	10%	No	F
Anxious (Generalized Anxiety Disorder)	5.73	4	35%	54%	10%	No	F
Apneic (Sleep Apnea)	2.50	2	8%	15%	**77%**	Yes	M^*^
Autistic (Autism Spectrum Disorder)	6.25	3	44%	50%	6%	No	M
Bipolar (Bipolar Disorder)	6.69	2.75	63%	31%	6%	No	F^*^
Borderline (Borderline Personality Disorder)	6.88	2.75	67%	25%	8%	No	F^*^
Bulimic (Bulimia Nervosa)	6.21	3.75	52%	40%	8%	No	F
Crazy	7.94	2	**85%**	8%	6%	Yes	M^*^
Delusional (Delusional Disorder)	6.63	2.75	60%	27%	13%	No	M^*^
Depersonalized/Derealized (Depersonalization/Derealization Disorder)	4.48	3	21%	38%	42%	No	F^*^
Depressive (Major Depressive Disorder)	5.65	3	40%	38%	23%	No	F
Diogenes (Hoarding Disorder)	6.83	4	65%	23%	13%	No	M^*^
Disruptive (Conduct Disorder)	5.60	3.75	40%	38%	23%	No	M
Dissociated (Dissociative Identity Disorder)	5.15	4	33%	38%	29%	No	F^*^
Drug Addict (Substance Use Disorder)	8.13	1.75	**90%**	10%	0%	Yes	M
Dysmorphic (Body Dysmorphic Disorder)	4.94	3	25%	48%	27%	No	F^*^
Dysphoric (Dysphoria)	4.54	4	21%	44%	35%	No	F^*^
Dysthymic (Persistent Depressive Disorder—Dysthymia)	5.50	2.75	29%	52%	19%	No	F
Explosive (Intermittent Explosive Disorder)	5.73	3.75	42%	38%	21%	No	M
Frigid (Interest/Arousal Disorder)	5.71	5	46%	21%	33%	No	F
Heroin Addict (Opioid Use Disorder)	7.75	2	**85%**	6%	8%	Yes	M
Hyperactive/ADHD (Attention-Deficit/Hyperactivity Disorder)	5.31^†^	4	35%	33%	31%	No	M
Hypochondriac (Illness Anxiety Disorder)	5.96	3.75	50%	35%	15%	No	F^*^
Hysterical (Histrionic Personality Disorder)	7.19^†^	2	**77%**	15%	8%	Yes	F
Kleptomaniac (Kleptomania)	6.33	3.75	54%	31%	15%	No	F^*^
Malingerer (Factitious Disorder)	5.17	4	29%	42%	29%	No	F^*^
Maniac (Obsessive-Compulsive Disorder)	6.13	3	50%	33%	17%	No	F^*^
Narcoleptic (Narcolepsy)	3.40	4.75	13%	23%	65%	No	M^*^
Negativistic (Oppositional Defiant Disorder)	4.81	3.75	25%	46%	29%	No	M^*^
Nymphomaniac (Compulsive Sexual Behavior Disorder, ICD-11)	6.88	3.5	67%	23%	10%	No	F
Panicky (Panic Disorder)	5.00	3.75	29%	46%	25%	No	F
Paranoid (Paranoid Disorder)	7.27	3	69%	29%	2%	No	M^*^
Phobic (Specific Phobia)	4.77	3	19%	52%	29%	No	F^*^
Psychotic (Brief Psychotic Disorder)	7.31	3	73%	23%	4%	No	M
Pyromaniac (Pyromania)	7.19	2.75	**75%**	17%	8%	Yes	M
Retarded (Intellectual Disability)	8.94	0	**100%**	0%	0%	Yes	M^*^
Ruminator (Rumination Disorder)	4.40	3.75	19%	46%	35%	No	F^*^
Schizoid (Schizoid Personality Disorder)	6.65^†^	4	58%	29%	13%	No	M
Schizophrenic (Schizophrenia)	7.96	2	**85%**	13%	2%	Yes	M
Somatizer (Somatic Symptom Disorder)	5.06	4	33%	35%	31%	No	F^*^
Stressed (Acute Stress Disorder)	3.90^†^	3.75	21%	27%	52%	No	F^*^
Stutterer (Fluency Disorder—tuttering)	5.50	4.75	40%	35%	25%	No	M^*^
Trichotillomaniac (Trichotillomania)	4.08	3.75	17%	38%	46%	No	F^*^

^*^In labels where “both sexes” was selected by most participants, the final sex attribution was assigned to the category (male or female) with the highest percentage among the remaining responses. ^†^Statistically significant differences between health professionals and service users (*p* < 0.05). Direction of group differences is reported in Supplementary Table S1. Values in bold indicate categories that reached consensus according to the predefined RAND/UCLA criteria (≥ 75% of ratings within the same category and IQR ≤ 3).

Participants also provided qualitative feedback, which was considered and used to refine or add new terms for subsequent rounds.

In addition, comparisons between health professionals and mental health service users revealed statistically significant differences in the perceived level of stigmatization for four diagnostic labels with effect sizes ranging from small to moderate (see Supplementary Table S1). Specifically, service users rated *schizoid* as significantly more stigmatizing than professionals (*p* = 0.022), whereas professionals assigned higher stigmatization scores than service users to *hyperactive*/*ADHD* (*p* = 0.020), *hysterical* (*p* = 0.049), and *stressed* (*p* = 0.019). No statistically significant differences between groups were observed for the remaining diagnostic labels (*p* > 0.05).

Drawing on participants' feedback, the research team reviewed all proposed modifications, additions, and deletions during an internal consensus meeting. As a result, additional terms were included to improve clarity. Specifically, *obsessive* was added together with *maniac*. Likewise, *vigorexic* was included together with *dysmorphic*, and *histrionic* with *hysterical*, even though the latter had already reached consensus during the first round, as participants' comments and the research team's subsequent discussion indicated that these labels conveyed distinct stigmatizing connotations and should therefore be evaluated separately. Participants also suggested including an additional term related to substance use disorders (*drug abuser*-*toxicómano/a* in Spanish). As a result, 15 new labels were added for evaluation in the next round based on participants' comments from Round 1. In addition, nine labels (*despersonalized, diogenes, dysphoric, narcoleptic, panicky, ruminator, schizoid, somatizer* and *trichotillomaniac*) were removed after the first round because participants indicated that these terms were difficult to interpret or not representative of commonly used diagnostic language.

### 3.2 Round 2

During the second Delphi round, 28 participants completed the questionnaire: 20 health professionals and 8 service users. The professionals represented diverse disciplines, including Mental health nursing (*n* = 3), Clinical psychology (*n* = 2), Primary care medicine (*n* = 3), Social work (*n* = 3), Psychology (*n* = 4), Primary care nursing (*n* = 1), Occupational therapy (*n* = 1), Psychiatry (*n* = 2), and General nursing (*n* = 1).

Of the 44 items assessed, eight labels reached consensus as highly stigmatizing (e.g., *paranoid, psychotic, unbalanced*, and *drug abuser*), whereas the remaining labels did not meet the agreement threshold (see Table 3).

**Table 3:** Round 2 results (*N* = 28).

Label			Level of stigmatization				
	*M*	IQR	High (7–9)	Medium (4–6)	Low (1–3)	Consensus	Sex-related applicability
Agoraphobic (Agoraphobia)	4.64	2.75	14.29%	57.14%	28.57%	No	
Alcoholic (Alcohol Use Disorder)	7.00	2	64.29%	32.14%	3.57%	No	
Amnesic (Dissociative Amnesia)	4.04	2	10.71%	39.29%	50%	No	
Anorexic (Anorexia Nervosa)	6.64	2.75	64.29%	32.14%	3.57%	No	
Anxious (Generalized Anxiety Disorder)	5.00	3.75	25%	46.43%	28.57%	No	
Autistic (Autism Spectrum Disorder)	6.43	2.75	64.29%	25%	10.71%	No	
Bipolar (Bipolar Disorder)	6.64	2	53.57	42.86%	3.57%	No	
Borderline (Borderline Personality Disorder)	7.07	2	71.43	25%	3.57%	No	
Bulimic (Bulimia Nervosa)	6.18	3	42.86%	50%	7.14%	No	
Delusional (Delusional Disorder)	7.18	2.5	**75%**	17.86%	7.14%	Yes	
Depressive (Major Depressive Disorder)	5.57	1.75	25%	60.71%	14.29%	No	
Disruptive (Conduct Disorder)	5.18	2.75	25%	60.71%	14.29%	No	
Dissociated (Dissociative Identity Disorder)	5.29	3.75	28.57%	46.43%	25%	No	
Dysmorphic (Body Dysmorphic Disorder)	4.75	3	14.29%	57.14%	28.57%	No	
Dysthymic (Persistent Depressive Disorder—Dysthymia)	5.04	2	21.43%	57.14%	21.43%	No	
Explosive (Intermittent Explosive Disorder)	5.54	3	42.86%	39.29%	17.86%	No	
Frigid (Interest/Arousal Disorder)	6.36	4	53.57%	32.14%	14.29%	No	
Hyperactive/ADHD (Attention-Deficit/Hyperactivity Disorder)	5.21	3.5	25%	50%	25%	No	
Hypochondriac (Illness Anxiety Disorder)	5.75	3	39.29%	46.43%	14.29%	No	
Kleptomaniac (Kleptomania)	6.32	3	46.43%	42.86%	10.71%	No	
Malingerer (Factitious Disorder)	5.96	3.5	35.71%	57.14%	7.14%	No	
Maniac (Obsessive-Compulsive Disorder)	5.75	4	42.86%	42.86%	14.29%	No	
Negativistic (Oppositional Defiant Disorder)	4.96	4	32.14%	35.71%	32.14%	No	
Nymphomaniac (Compulsive Sexual Behavior Disorder, ICD-11)	6.75	3	60.71%	25%	14.29%	No	
Paranoid (Paranoid Disorder)	7.11	1	**78.57%**	14.29%	7.14%	Yes	
Phobic (Specific Phobia)	4.96	2.75	21.43%	53.57%	25%	No	
Psychotic (Brief Psychotic Disorder)	7.39	2	**82.14%**	14.29%	3.57%	Yes	
Stressed (Acute Stress Disorder)	3.89	2.75	14.29%	32.14%	53.57%	No	
Stutterer (Fluency Disorder—Stuttering)	5.54	3.5	53.57%	14.29%	32.14%	No	
Newly added terms *(n=15)*							
Antisocial (Antisocial Personality Disorder)	5.93	3.75	53.57%	28.57%	17.86%	No	M
Cocaine Addict (Cocaine Use Disorder)	7.25	2.75	**75%**	25%	0.00%	Yes	M
Demented (Neurocognitive Disorder)	7.04	2.5	**75%**	14.29%	10.71%	Yes	F
Dependent (Dependent Personality Disorder)	5.32	3	32.14%	57.14%	10.71%	No	F
Disturbed	7.75	1.75	**82.14%**	14.29%	3.57%	Yes	F^*^
Drug abuser (Substance Use Disorder)	7.64	2	**89.29%**	7.14%	3.57%	Yes	M
Dyslexic (Specific Learning Disorder with Impairment in Reading)	4.43	3.75	21.43%	46.43%	32.14%	No	M^*^
Fetishistic (Fetishistic Disorder)	5.54	4	46.43%	25%	28.57%	No	M
Histrionic (Histrionic Personality Disorder)	6.18	3.5	53.57%	32.14%	14.29%	No	F
Manic (Bipolar I Disorder)	6.75	2.75	67.86%	28.57%	3.57%	No	F^*^
Narcissistic (Narcissistic Personality Disorder)	6.39	3	60.71%	32.14%	7.14%	No	M
Obsessive (Obsessive-Compulsive Disorder)	5.50	3	35.71%	42.86%	21.46%	No	M^*^
Unbalanced	7.54	2	**82.14%**	10.71%	7.14%	Yes	F^*^
Vigorexic (Body Dysmorphic Disorder)	5.25	3.75	28.57%	46.43%	25%	No	M
Voyeuristic (Voyeuristic Disorder)	6	3.75	53.57%	25%	21.43%	No	M

^*^In labels where “both sexes” was selected by most participants, the final sex attribution was assigned to the category (male or female) with the highest percentage among the remaining responses. Values in bold indicate categories that reached consensus according to the predefined RAND/UCLA criteria (≥ 75% of ratings within the same category and IQR ≤ 3).

Regarding sex-related applicability for the new terms, 46% of the labels were judged as more frequently associated with men (e.g., *antisocial, cocaine addict, fetishistic*, and *vigorexic*), whereas 20% were linked to women (e.g., *demented* and *histrionic*). The rest of the labels were perceived as equally applicable to both sexes.

Participants were also invited to provide additional qualitative feedback on the revised and newly added terms to inform subsequent Delphi rounds.

### 3.3 Round 3

In the third and final Delphi round, responses were obtained from 26 participants, including 22 health professionals and related specialists and 4 service users. The professionals' qualifications were as follows: Mental health nursing (*n* = 4), Clinical psychology (*n* = 2), Primary care medicine (*n* = 2), Social work (*n* = 3), Psychology (*n* = 4), Primary care nursing (*n* = 1), Occupational therapy (*n* = 1), and Psychiatry (*n* = 1).

After removing the labels that had reached consensus in the previous round, the list for the final round included 36 diagnostic terms.

Participants identified the following four labels as highly stigmatizing: *alcoholic, bipolar, manic*, and *nymphomaniac*. The remaining labels did not reach the predefined consensus threshold, despite three iterative rounds of evaluation (Table 4).

**Table 4:** Round 3 results (*N* = 26).

Label			Level of stigmatization			
	*M*	IQR	High (7–9)	Medium (4–6)	Low (1–3)	Consensus
Agoraphobic (Agoraphobia)	5.11	2	22.22%	59.26%	18.52%	No
Alcoholic (Alcohol Use Disorder)	7.48	2	**77.78%**	18.52%	3.70%	Yes
Amnesic (Dissociative Amnesia)	4.59	3	14.81%	44.44%	40.74%	No
Anorexic (Anorexia Nervosa)	6.74	2	59.26%	37.04%	3.70%	No
Anxious (Generalized Anxiety Disorder)	5.93	3	40.74%	48.15%	11.11%	No
Antisocial (Antisocial Personality Disorder)	7	4	70.37%	22.22%	7.41%	No
Autistic (Autism Spectrum Disorder)	6.37	3	55.56%	33.33%	11.11%	No
Bipolar (Bipolar Disorder)	7.15	1	**77.78%**	18.52%	3.70%	Yes
Borderline (Borderline Personality Disorder)	7.04	2	70.37%	25.93%	3.70%	No
Bulimic (Bulimia Nervosa)	6.37	3	44.44%	55.56%	0.00%	No
Dependent (Dependent Personality Disorder)	5.63	2	37.04%	44.44%	18.52%	No
Depressive (Major Depressive Disorder)	6.07	2	48.15%	40.74%	11.11%	No
Dyslexic (Specific Learning Disorder with Impairment in Reading)	4.78	3	22.22%	40.74%	37.04%	No
Dysmorphic (Body Dysmorphic Disorder)	5.11	4	25.93%	40.74%	33.33%	No
Dissociated (Dissociative Identity Disorder)	5.44	3	29.63%	48.15%	22.22%	No
Disruptive (Conduct Disorder)	5.85	2	51.85%	29.63%	18.52%	No
Dysthymic (Persistent Depressive Disorder—Dysthymia)	5.41	3	25.93%	55.56%	18.52%	No
Explosive (Intermittent Explosive Disorder)	6.33	3	55.56%	33.33%	11.11%	No
Fetishistic (Fetishistic Disorder)	5.93	5	37.04%	40.74%	22.22%	No
Frigid (Interest/Arousal Disorder)	6.22	3	51.85%	33.33%	14.81%	No
Hyperactive/ADHD (Attention-Deficit/Hyperactivity Disorder)	5.78	4	40.74%	37.04%	22.22%	No
Hypochondriac (Illness Anxiety Disorder)	6.15	2	40.74%	48.15%	11.11%	No
Histrionic (Histrionic Personality Disorder)	6.63	2	62.96%	29.63%	7.41%	No
Kleptomaniac (Kleptomania)	6.89	3	66.67%	25.93%	7.41%	No
Malingerer (Factitious Disorder)	6.37	4	48.15%	37.04%	14.81%	No
Maniac (Obsessive-Compulsive Disorder)	6.37	3	48.15%	44.44%	7.41%	No
Manic (Bipolar I Disorder)	7.33	2	**77.78%**	19.52%	3.70%	Yes
Narcissistic (Narcissistic Personality Disorder)	6.85	2	70.37%	22.22%	7.41%	No
Negativistic (Oppositional Defiant Disorder)	5.59	3	37.04%	40.74%	22.22%	No
Nymphomaniac (Compulsive Sexual Behavior Disorder, ICD-11)	7.30	2	**77.78%**	18.52%	3.70%	Yes
Obsessive (Obsessive-Compulsive Disorder)	6.04	2	48.15%	40.74%	11.11%	No
Phobic (Specific Phobia)	5.26	4	33.33%	40.74%	25.93%	No
Stressed (Acute Stress Disorder)	4.33	2	18.52%	33.33%	48.15%	No
Stutterer (Fluency Disorder—Stuttering)	5.81	5	51.85%	22.22%	25.93%	No
Vigorexic (Body Dysmorphic Disorder)	5.07	3	25.93%	51.85%	22.22%	No
Voyeuristic (Voyeuristic Disorder)	5.96	3	44.44%	37.04%	18.52%	No

Values in bold indicate categories that reached consensus according to the predefined RAND/UCLA criteria (≥ 75% of ratings within the same category and IQR ≤ 3).

### 3.4 Main findings

Regarding participation across rounds, 48 participants contributed data in the first round, 28 in the second round, and 26 in the final round, corresponding to an overall attrition rate of 45.8% from the first to the third round.

To explore the potential impact of attrition, sociodemographic characteristics of participants who completed all Delphi rounds were compared with those who withdrew during the process. No statistically significant differences were observed in sex, age, or educational level (all p > 0.05), suggesting that attrition was not systematically associated with the characteristics assessed (see Supplementary Table S2).

Across the three-round Delphi process, 61 distinct terms were evaluated, including the initial DSM-5–derived set and additional terms suggested by participants.

Of the 61 labels evaluated, 20 reached consensus (32.78%). Among these, 95% were classified as highly stigmatizing and 5% as low in stigma, while none reached consensus for a moderate level of stigmatization. In terms of sex-based applicability, 51% of the labels were perceived as applicable to both sexes, 31% as more applicable to men, and 18% as more applicable to women.

#### 3.4.1 Participants comments and impressions

In addition to the quantitative ratings, participants provided open-text responses to qualitative questions included in each round of the Delphi. They were invited to comment on the labels evaluated, explain the reasoning behind their ratings, and flag any terms they considered ambiguous, unfamiliar, or particularly charged. A thematic analysis of these responses across all three rounds identified five recurrent themes that could help to understand both the patterns of consensus achieved and the widespread non-consensus observed: (1) contextual variability in stigma perception, whereby the same label was perceived as affectionate or deeply stigmatizing depending on the context of use; (2) terminological unfamiliarity and semantic confusion, whereby some labels were poorly recognized or confused with related terms—for example, *schizoid* vs. *schizophrenic*; (3) social overuse and misuse of labels, whereby colloquial terms accumulated an additional stigmatizing layer beyond their clinical referent; (4) moral attribution and perceived responsibility, with addiction-related labels consistently eliciting rejection on the basis of perceived personal choice; and (5) the linguistic framing of diagnosis, whereby defining a person by their diagnosis (“*he is schizophrenic”*) was perceived as considerably more stigmatizing than describing a temporary state (“*he has schizophrenia”*).

## 4 Discussion

This study assessed the perceived stigma associated with a broad range of terms, mainly derived from DSM-5 diagnostic labels, using a Delphi approach that integrated perspectives from both health professionals and mental health service users. A central finding of the Delphi process was the limited level of consensus regarding stigma: approximately 67% of the labels failed to reach the predefined 75% agreement threshold, even after three iterative rounds.

The decision to conclude the process was made in accordance with established methodological recommendations, which typically advise two to three iterative rounds to balance stability of responses with participant burden (Fitch, 2001). Importantly, the lack of consensus itself constitutes a meaningful finding. It raises the possibility that stigma toward MDs is not a unitary construct, but an emergent phenomenon shaped by individual experience, professional background, and sociocultural context (Varaona et al., 2024). This is consistent with previous research showing that stigma is a dynamic construct influenced by familiarity, clinical background, and lived experience (Gambini et al., 2019; Ghuloum et al., 2022; Sastre-Rus et al., 2020).

Although many labels did not reach the predefined consensus threshold, several clear patterns emerged across the Delphi rounds. Terms such as *borderline, kleptomaniac, narcissist*, and *hysterical* consistently received high stigma ratings, even when they narrowly fell short of the agreement criterion. In contrast, labels such as *stressed* elicited more heterogeneous evaluations. Many people interpreted this term as a normative or situational state rather than a mental disorder (Piao et al., 2024), and prior research suggests that, in certain contexts, stress-related labels may even be associated with effort, engagement, or aspirational social signaling (Bellezza et al., 2017). These examples illustrate how frequency of use and social framing can attenuate perceived stigma without eliminating underlying ambivalence. Nevertheless, a small number of labels achieved clear and consistent consensus as highly stigmatizing across the Delphi rounds. The labels *paranoid, psychotic*, and *schizophrenic* received particularly high stigma ratings, reinforcing extensive evidence that psychotic disorders remain among the most socially discredited mental health conditions (Anglin et al., 2014; Caqueo-Urízar et al., 2022; Manago and Mize, 2024). Notably, all labels related to substance use disorders also reached consensus as highly stigmatizing, consistent with previous research documenting the persistent stigma attached to addiction-related terminology (Nascimento and Leão, 2019).

Other highly stigmatizing labels may be interpreted in light of broader stigma mechanisms documented in the literature. For instance, *pyromaniac* was rated as highly stigmatizing, consistent with psychiatric literature emphasizing the fear associated with intentional fire-setting and its perceived risk to life and property (Sugarman and Dickens, 2009). Similarly, the strong stigmatization of *demented* aligns with extensive evidence documenting negative stereotypes, social distancing, and dehumanizing attitudes toward individuals with dementia (Herrmann et al., 2018; Low and Purwaningrum, 2020; Rewerska-Juśko and Rejdak, 2020). In contrast, *apneic* was consistently rated as low in stigma, possibly reflecting its perception as a medical rather than psychiatric condition. Research indicates that highly medicalized conditions tend to evoke lower stigma than disorders framed in moral or behavioral terms (Weiner et al., 1988).

Colloquial or derogatory terms *crazy* and *hysterical* were also unanimously perceived as highly stigmatizing. Previous research has documented the widespread use of these expressions in everyday language as markers of deviance and social discredit (Martínez León, 2023; Ruano Fosch, 2020). In addition, clinical labels as *retarded, bipolar*, and *manic*, elicited high stigma ratings. These findings are consistent with research indicating that diagnoses perceived as involving impaired functioning or behavioral instability often evoke stronger stigmatizing responses (Hawke et al., 2013; Scior et al., 2020). In this same vein, the label *unbalanced* was also strongly stigmatized. Although this specific term appears to have received little direct empirical attention, prior research suggests that perceptions of instability or unpredictability constitute central drivers of mental illness stigma (Kenny et al., 2018). Taken together, these findings align with research suggesting that stigma is shaped less by diagnostic categories themselves than by perceptions of danger, unpredictability, and impaired volitional control (Link et al., 1999; Manago and Mize, 2024).

Beyond perceptions of instability, stigma may also be reinforced by gendered moral judgments. In this line, the label *nymphomaniac* elicited high levels of stigmatization, consistent with research documenting its historically negative and gendered connotations related to female sexuality (Combridge and Lastella, 2022; Kowalewska et al., 2025).

The thematic analysis of participants' qualitative feedback provides an additional context for these findings. In particular, the predominance of contextual variability could help to explain why many labels failed to reach consensus, suggesting that their stigmatizing potential is influenced by situational and social factors rather than being an inherent property of the label itself (Manago and Mize, 2024). Likewise, the theme of moral attribution resonates with attribution-based models of stigma, which propose that conditions perceived as controllable tend to elicit stronger blame and social rejection (Foster and O'Mealey, 2022). This perspective may help contextualize the consistently high stigma ratings observed for addiction-related labels across the Delphi rounds.

Comparisons between health professionals and mental health service users revealed statistically significant differences in perceived stigmatization for only four of the 61 labels evaluated (*schizoid, hysterical, hyperactive/ADHD*, and *stressed*). The limited number of discrepancies may reflect a largely shared framework in how diagnostic labels are perceived across groups. These differences may therefore reflect nuanced variations in sensitivity to particular diagnostics or colloquial terms rather than fundamentally different stigma schemas (Jacobs and Quinn, 2022). Although causal interpretations cannot be drawn, several factors may help contextualize these differences. Professional socialization and sustained clinical exposure may lead health professionals to interpret certain labels primarily through a diagnostic lens (Bohman, 2023), whereas service users may be more attuned to their everyday or pejorative use (Eads et al., 2021). Conversely, heightened professional awareness of stigma surrounding specific terms may also increase sensitivity to labels that have a strong history of misuse or trivialization in public discourse (Raney et al., 2021). These findings should be interpreted in light of the exploratory nature of the comparisons between stakeholder groups and the relatively small number of labels for which significant differences were observed.

This study also sheds light on gender-related patterns in the perceived applicability of several diagnostic labels. Notable sex-based differences emerged in how certain terms were perceived. Labels related to eating disorders and related concepts—such as *anorexic, bulimic, hysterical*, or dependency-related terms—were predominantly perceived as feminine, whereas labels such as *pyromaniac, antisocial*, or *narcissist* were more frequently associated with men. These patterns are broadly consistent with previous literature documenting gendered perceptions of mental health conditions and personality traits. For example, eating disorders and certain neurotic or hysterical tropes have historically been more frequently attributed to women (Alcañiz et al., 2015; Tasca et al., 2012; Tarissi De Jacobis et al., 2025; Villar Del Saz Bedmar and Baile Ayensa, 2023), whereas antisocial behavior, narcissistic traits, and substance-use-related labels (e.g., *cocaine* or *drug addict*) are more commonly stereotyped as masculine (Grijalva et al., 2015; Cantos Vicent, 2020).

Some findings, however, appear less documented in previous research. Labels such as *voyeuristic, vigorexic*, or *fetishist* were predominantly perceived as male-oriented, whereas *frigid* was more frequently associated with women. Although the reasons underlying these perceptions cannot be established from the present data, previous literature has linked muscularity and bodybuilding to masculine ideals (Ganson et al., 2025; Murray et al., 2013), and has documented the historical pathologization of low sexual desire in women (Van Anders et al., 2022). Future research should examine how the same labels are perceived when applied to individuals of different genders.

In this context, these patterns raise the possibility that stigma may be amplified when diagnostic labels intersect with gender norms or expectations (Link and Phelan, 2001), potentially producing forms of compounded or “double” stigmatization (Maloney et al., 2024). Similar dynamics have been observed in other health contexts. For instance, men diagnosed with conditions traditionally associated with women, such as breast cancer, often experience compounded stigma due both to the illness itself and to the perceived violation of gender norms (Lehe et al., 2025; Levin-Dagan and Baum, 2021). Such internalized stereotypes may discourage help-seeking and hinder disclosure of health problems (Capuano et al., 2025; MacLean et al., 2015).

An additional consideration is the linguistic structure of Spanish, a language in which grammatical gender is morphologically marked. As a result, the same diagnostic category may evoke different associations depending on whether it is expressed in its masculine or feminine form (Bender et al., 2016, 2018). Moreover, variation within the same diagnostic domain—for example, labels such as *drug addict* and *drug abuser—*may carry distinct connotations despite referring to overlapping clinical phenomena. Overall, these observations highlight that perceptions of stigma may be shaped not only by the diagnostic concept itself, but also by linguistic form, lexical choice, and the cultural salience of specific terms (O'Connor et al., 2022).

Overall, the present findings are consistent with the central role of language in the construction and perpetuation of mental health stigma (Mascayano et al., 2015; Vicario Cañas and Moral Jiménez, 2017). For both researchers and clinicians, these results caution against assuming that diagnostic labels carry fixed or universally shared meanings, reinforcing the need for context-sensitive and reflexive use of mental health terminology.

## 5 Implications

The present study has primarily methodological implications. First, the findings indicate that diagnostic labels cannot be assumed to function as stigma-neutral or interchangeable linguistic stimuli. Even when derived from standardized classification systems, labels differ substantially in their perceived stigmatizing potential. For experimental research, this underscores the importance of empirically characterizing stimuli before their use in affective, cognitive, or neurophysiological paradigms. Failure to do so may introduce uncontrolled variance related to stigma rather than to the intended construct.

Second, the high rate of non-consensus highlights the multidimensional and context-dependent nature of stigma judgments. Rather than representing a methodological shortcoming, the 41 labels that failed to reach consensus may constitute a valuable resource for future research. Researchers may choose to (a) incorporate the degree of consensus associated with each label as a continuous indicator of perceived stigma, (b) use these labels as stimuli for examining individual variability in stigma perceptions (i.e., serving as standardized “ambiguous anchors” in baseline-vs-treatment experimental paradigms), or (c) explore whether broader dimensions underlying stigma judgments can be identified through subsequent empirical work. Such approaches may contribute to a more nuanced understanding of how diagnostic terminology is perceived across different populations and contexts.

Researchers designing experiments using diagnostic terminology should therefore consider whether to (a) pre-select stimuli based on consensual stigma levels, (b) measure perceived stigma at the individual level and model it statistically, or (c) explicitly manipulate stigma as an experimental factor.

Third, the study provides a systematically evaluated pool of diagnostic terms that may serve as a foundation for subsequent affective validation. By combining structured expert–stakeholder consensus procedures with predefined agreement thresholds, this work contributes to the development of more rigorously characterized linguistic stimulus sets. Such characterization enhances transparency, replicability, and interpretability in studies examining stigma perception, or language-related biases.

Finally, the methodological strategy adopted in this study—combining Delphi procedures with predefined RAND-based consensus criteria—illustrates a structured approach for refining stimulus sets prior to experimental use. Such procedures may be particularly valuable in research areas where socially sensitive terminology is involved and where the stigmatizing potential of linguistic stimuli cannot be assumed *a priori*. By systematically characterizing stimuli before their inclusion in experimental paradigms, researchers may reduce uncontrolled variability and enhance the interpretability of findings.

## 6 Limitations

Limitations include the smaller representation of service users relative to professionals, the culturally specific Spanish linguistic context, and the process used for the selection and refinement of labels, which may not fully capture the diversity of stigma-relevant terminology. In addition, the iterative addition and removal of labels across Delphi rounds introduced some heterogeneity into the evaluated stimulus pool. Although these modifications were guided by participant feedback and formed part of the exploratory refinement process, they should be considered when interpreting round-specific consensus estimates.

Attrition across Delphi rounds represents an additional limitation. Although participant loss is common in iterative consensus procedures, the reduction in panel size across rounds may have influenced the representativeness of the final sample and the stability of consensus judgments. In particular, differential attrition among mental health service users resulted in their reduced representation in the later rounds, which may have limited the extent to which experiential perspectives were reflected in the final ratings. To assess the potential impact of selection bias, sociodemographic characteristics of participants who completed the study were compared with those who withdrew during the process. and no significant differences were observed in sex, age, or educational level, suggesting that attrition was not systematically associated with the characteristics assessed.

Also, the higher representation of professional profiles and participants with advanced educational levels may have inadvertently skewed the consensus toward more formalized, abstract, or clinically conventional conceptualizations of the labels. Conversely, the nuanced perspectives of service users—which are often rooted in lived experience, emotional resonance, and pragmatic usage—might be underrepresented in the final consensus thresholds. Consequently, items that failed to reach consensus may not inherently lack validity; rather, they might reflect a conceptual tension or a divergence in semantic interpretation between professional-academic discourse and the experiential reality of service users. Future validation studies should employ stratified sampling to systematically contrast how these distinct stakeholder groups decode the target terminology.

This imbalance may represent a potential limitation, as variations in educational background could influence familiarity with diagnostic terminology, interpretative frameworks, or exposure to anti-stigma narratives. Nevertheless, the possibility that unmeasured factors influenced continued participation cannot be excluded and should be considered when interpreting the findings.

Despite these limitations, the study offers valuable insight into how diagnostic labels are perceived in terms of stigma and gender associations, and it provides a foundation for future research in this area. Future studies should examine whether these patterns are replicated in samples with different sociocultural and educational profiles.

## 7 Conclusion

This preregistered Delphi study provides a systematic characterization of the perceived stigmatizing potential of mental health-related labels. The results revealed substantial variability in stigma judgments, with consensus achieved for only a limited subset of terms after three iterative rounds. These findings highlight that diagnostic terminology should not be assumed to carry uniform social meanings, and that perceptions of stigma may vary according to context, individual experience, and sociocultural background.

Methodologically, the study offers a transparent procedure for characterization and refinement of mental health-related terminology trough a structured consensus process involving both health professionals and mental health users. The resulting dataset provides an empirically informed resource for future research examining stigma, language, and the social perception of diagnostic labels.

## Data Availability

The raw data supporting the conclusions of this article will be made available by the authors, without undue reservation.
