# Supplementary material for: Perceptions of mental health stigma toward diagnostic labels among health professionals and mental health users: a Delphi study
Source: Front Psychol. 2026 Aug 7;17:1863313. doi: 10.3389/fpsyg.2026.1863313 (PMC13493301; doi:10.3389/fpsyg.2026.1863313)
Supplement: Supplementary file 1 [file Table_1.docx]

Supplementary Table 1. *Comparison of labels between health professionals and mental health users.*

| **Label** | **Health Professionals** | **Mental Health Users** | **Test** | ***p*** | **Effect Size** |
| --- | --- | --- | --- | --- | --- |
| Agoraphobic (Agoraphobia) | 4.91 | 4.36 | 193 | .300 | .191 |
| Alcoholic (Alcohol Use Disorder) | 7.47 | 6.21 | 172 | .128 | .277 |
| Amnesic (Dissociative Amnesia) | 3.50 | 4.43 | 181 | .191 | .241 |
| Anorexic (Anorexia Nervosa) | 6.24 | 6.14 | 235 | .954 | .012 |
| Anxious (Generalized Anxiety Disorder) | 5.88 | 5.36 | 195 | .329 | .180 |
| Apneic (Sleep Apnea) | 2.44 | 2.64 | 209 | .491 | .121 |
| Autistic (Autism Spectrum Disorder) | 6.41 | 5.86 | 199 | .370 | .166 |
| Bipolar (Bipolar Disorder) | 6.44 | 7.29 | 164 | .090 | .310 |
| Borderline (Borderline Personality Disorder) | 6.71 | 7.29 | 185 | .223 | .222 |
| Bulimic (Bulimia Nervosa) | 6.26 | 6.07 | 231 | .871 | .031 |
| Crazy | 7.88 | 8.07 | 223 | .709 | .063 |
| Delusional (Delusional Disorder) | 6.65 | 6.57 | 229 | .836 | .039 |
| Depersonalized / Derealized (Depersonalization/ Derealization Disorder) | 4.21 | 5.14 | 181 | .190 | .241 |
| Depressive (Major Depressive Disorder) | 5.76 | 5.36 | 207 | .475 | .132 |
| Diogenes (Hoarding Disorder) | 6.65 | 7.29 | 206 | .454 | .136 |
| Disruptive (Conduct Disorder) | 5.62 | 5.57 | 233 | .909 | .023 |
| Dissociated (Dissociative Identity Disorder) | 4.82 | 5.93 | 181 | .196 | .239 |
| Drug Addict (Substance Use Disorder) | 8.21 | 7.93 | 228 | .803 | .044 |
| Dysmorphic (Body Dysmorphic Disorder) | 4.76 | 5.36 | 203 | .428 | .147 |
| Dysphoric (Dysphoria) | 4.59 | 4.43 | 225 | .766 | .056 |
| Dysthymic (Persistent Depressive Disorder – Dysthymia) | 5.56 | 5.36 | 227 | .808 | .046 |
| Explosive (Intermittent Explosive Disorder) | 5.50 | 6.29 | 204 | .436 | .145 |
| Frigid (Interest/Arousal Disorder) | 5.76 | 5.57 | 224 | .756 | .058 |
| Heroin Addict (Opioid Use Disorder) | 7.65 | 8.00 | 204 | .403 | .145 |
| Hyperactive / ADHD (Attention-Deficit/Hyperactivity Disorder) | 5.79 | 4.14 | 137 | .021* | .426 |
| Hypochondriac (Illness Anxiety Disorder) | 6.29 | 5.14 | 164 | .089 | .313 |
| Hysterical (Histrionic Personality Disorder) | 7.56 | 6.29 | 154 | .050* | .355 |
| Kleptomaniac (Kleptomania) | 6.15 | 6.79 | 209 | .512 | .121 |
| Malingerer (Factitious Disorder) | 5.44 | 4.50 | 182 | .200 | .237 |
| Maniac (Obsessive-Compulsive Disorder) | 6.12 | 6.14 | 233 | .909 | .023 |
| Narcoleptic (Narcolepsy) | 3.18 | 3.93 | 185 | .225 | .222 |
| Negativistic (Oppositional Defiant Disorder) | 4.94 | 4.50 | 213 | .566 | .107 |
| Nymphomaniac (Compulsive Sexual Behavior Disorder, ICD-11) | 6.79 | 7.07 | 231 | .871 | .031 |
| Panicky (Panic Disorder) | 4.76 | 5.57 | 193 | .301 | .191 |
| Paranoid (Paranoid Disorder) | 7.29 | 7.21 | 230 | .852 | .035 |
| Phobic (Specific Phobia) | 4.82 | 4.64 | 214 | .582 | .102 |
| Psychotic (Brief Psychotic Disorder) | 7.15 | 7.71 | 196 | .327 | .178 |
| Pyromaniac (Pyromania) | 6.85 | 8.00 | 165 | .090 | .306 |
| Retarded (Intellectual Disability) | 8.91 | 9.00 | 224 | .377 | .058 |
| Ruminator (Rumination Disorder) | 4.44 | 4.29 | 225 | .766 | .056 |
| Schizoid (Schizoid Personality Disorder) | 6.15 | 7.86 | 139 | .022* | .418 |
| Schizophrenic (Schizophrenia) | 7.79 | 8.36 | 184 | .179 | .229 |
| Somatizer (Somatic Symptom Disorder) | 5.26 | 4.57 | 195 | .331 | .180 |
| Stressed (Acute Stress Disorder) | 4.44 | 2.57 | 136 | .020* | .428 |
| Stutterer (Fluency Disorder) | 5.32 | 5.93 | 207 | .485 | .130 |
| Trichotillomaniac (Trichotillomania) | 3.68 | 5.07 | 154 | .056 | .352 |
| *Newly added terms (n=15)* |  |  |  |  |  |
| Antisocial (Antisocial Personality Disorder) | 5.96 | 5.80 | 52.5 | .784 | .086 |
| Cocaine Addict (Cocaine Use Disorder) | 7.61 | 5.60 | 21.5 | .029* | .626 |
| Demented (Neurocognitive Disorder) | 7.09 | 6.80 | 57 | 1 | .008 |
| Dependent (Dependent Personality Disorder) | 5.35 | 5.20 | 56 | .951 | .026 |
| Disturbed | 8.09 | 6.20 | 39.5 | .262 | .313 |
| Drug abuser (Substance Use Disorder) | 8.00 | 6.00 | 26.5 | .056 | .539 |
| Dyslexic (Specific Learning Disorder with Impairment in Reading) | 4.39 | 4.60 | 55.5 | .928 | .034 |
| Fetishistic (Fetishistic Disorder) | 5.83 | 4.20 | 37.5 | .236 | .347 |
| Histrionic (Histrionic Personality Disorder) | 6.09 | 6.60 | 47.5 | .562 | .173 |
| Manic (Bipolar I Disorder) | 6.87 | 6.40 | 57 | 1 | .008 |
| Narcissistic (Narcissistic Personality Disorder) | 6.52 | 5.80 | 51.5 | .737 | .104 |
| Obsessive (Obsessive-Compulsive Disorder) | 5.30 | 6.40 | 40 | .302 | .304 |
| Unbalanced | 7.65 | 7.00 | 48 | .571 | .165 |
| Vigorexic (Body Dysmorphic Disorder) | 5.09 | 6.00 | 43 | .396 | .252 |
| Voyeuristic (Voyeuristic Disorder) | 6.09 | 5.60 | 51.5 | .738 | .104 |

*Note*. **p ≤ 0,05.*
