# Supplementary material for: Perceptions of mental health stigma toward diagnostic labels among health professionals and mental health users: a Delphi study
Source: Front Psychol. 2026 Aug 7;17:1863313. doi: 10.3389/fpsyg.2026.1863313 (PMC13493301; doi:10.3389/fpsyg.2026.1863313)
Supplement: Supplementary file 2 [file Table_2.docx]

**Supplementary Table 2.** *Comparisons between retained vs attrited participants*

| **Variable** | **Retained**  **Participants**  **(n=26)** | **Attrited participants**  **(n=22)** | ***p*** |
| --- | --- | --- | --- |
| Age | 46.5 (39.2-55.0) | 44.5 (35.2-55.5) | .419 |
| Sex |  |  | .765 |
| Female | 16 (61.5) | 15 (68.2) |  |
| Male | 10 (38.5) | 7 (31.8) |  |
| Education level |  |  | .372 |
| Primary education | 0 (0) | 1 (4.5) |  |
| Secondary education | 1 (3.8) | 3 (13.6) |  |
| Higher education (undergraduate) | 10 (38.5) | 7 (31.8) |  |
